# Research on Tire Surface Damage Detection Method Based on Image Processing

**DOI:** 10.3390/s24092778

**Published:** 2024-04-26

**Authors:** Jiaqi Chen, Aijuan Li, Fei Zheng, Shanshan Chen, Weikai He, Guangping Zhang

**Affiliations:** 1 School of Automotive Engineering, Shandong Jiaotong University, Jinan 250357, China; achilleshelmet@163.com; 2 Research and Development Department, Shandong Wonderful Intelligent Technology Co., Ltd., Jinan 250101, China; chenss0608@126.com (S.C.); zhangguangping@shengquan.com (G.Z.); 3 School of Aeronautics, Shandong Jiaotong University, Jinan 250357, China; jiandanlcc@163.com

**Keywords:** bilateral filtering, multiscale, cluster, corner detection, histogram correlation

## Abstract

The performance of the tire has a very important impact on the safe driving of the car, and in the actual use of the tire, due to complex road conditions or use conditions, it will inevitably cause immeasurable wear, scratches and other damage. In order to effectively detect the damage existing in the key parts of the tire, a tire surface damage detection method based on image processing was proposed. In this method, the image of tire side is captured by camera first. Then, the collected images are preprocessed by optimizing the multi-scale bilateral filtering algorithm to enhance the detailed information of the damaged area, and the optimization effect is obvious. Thirdly, the image segmentation based on clustering algorithm is carried out. Finally, the Harris corner detection method is used to capture the “salt and pepper” corner of the target region, and the segmsegmed binary image is screened and matched based on histogram correlation, and the target region is finally obtained. The experimental results show that the similarity detection is accurate, and the damage area can meet the requirements of accurate identification.

## 1. Introduction

Wheel damage is one of the main manifestations of automobile damage, and the damage is mainly manifested in the tire tread and tire side. The tire side is the rubber layer between the tire bead and the tire shoulder attached to the side wall of the tire body, which plays a role in protecting the tire body [1]. When the wheel rotates rapidly, the side is also experiencing alternating motion of continuous recovery and deformation, so the quality requirements of the side of the tire are very high. Secondly, as it is not in contact with the ground, the force and wear are very small, and the thickness is thin. However, minor damage will lead to tire problems and tire blowout. Compared with the tread, tire side damage detection is more important. These defects will affect the quality of the tire and have potential dangers in the use of the tire [2,3]. At present, the detection of automobile tire damage is always manual, because of the variety of damage, the fact that the location is complicated, and the work efficiency is low. Therefore, it is of great significance to study the automatic detection of tire damage.

The early image detection technology has the limitation of hardware equipment and image precision, and the effect is not ideal when applied to automatic detection, especially in the detection of small targets with more interference. With the rapid development of the computer field, image detection technology has been widely used in many fields such as transportation and agriculture, and has achieved good results in solving the problem of small target detection. Sun He and Liu Shengbo et al. studied a 3D tire surface defect detection system based on machine vision. The system relies on the machine vision system to obtain the surface image of the detected tire, then creates a 3D model, determines the defect type, and finally realizes real-time automatic warning, providing an automatic detection scheme for tire manufacturers. The system integrates advanced technology, software and tools in order to achieve more efficient and reliable quality control in the production process and reduce inspection costs [4]. However, the system requires too much hardware and the modeling process is complicated. Zheng Bin, Luo Shan and Jiang Yincheng et al. combined machine vision and image processing technology to design a set of tire tread detection systems, and extracted accurate defect features through thresholding segmentation, defect labeling and morphological processing [5]. However, the detection object is limited to the tread with the pattern of image information distribution, which is obviously not applicable to the tread with complex information distribution and more irrelevant information.

The object detection algorithm based on deep learning exhibits high precision, robustness, scalability and real-time performance, making it a widely adopted approach in research [6,7,8,9]. In 2022, Gu Haijun and Chen Sheng et al. employed a visual image annotator to mark the features of cut-tire images, which were then presented as coordinate-based marked images. Subsequently, the Mask R-CNN network was utilized for adaptive training using a prepared training set. Finally, the trained model was evaluated through a test set [6]. In 2023, Sun Yingwei et al. from Qingdao University of Science and Technology proposed two tire defect detection methods based on deep learning technology incorporating semantic segmentation and an attention mechanism [7]. Zhao Mengmeng et al. introduced a lightweight tire defect detection method that leverages an attention mechanism along with proposing a multi-scale self-attention feature-enhancement module [8]. Yang Shihao et al. designed a Depth-wise Feature Pyramid Network (DWFPN), which is founded on Depth-wise convolution principles [9]. For the aforementioned series of methods aiming to reduce data volume within neural network feature maps, after passing through the convolution layer(s), input image size undergoes significant reduction while small targets occupying minimal area proportions may even vanish entirely from these feature maps. Consequently, this poses challenges in extracting features from small targets and distinguishing them amidst interference.

Aimed at the problems of the above algorithms, this paper studies a tire tread damage and scratch detection method based on image processing. The algorithm can be applied to the automatic detection of tire surface (mainly tire side) scratch damage, and has the advantages of high accuracy, flexibility and adaptability. In this paper, the difficulty of visual detection of tire scratches and damage based on image processing is to distinguish and capture the damage target area and exclude the non-target area. Then, the main flow of image processing algorithms and the optimization principle of their respective algorithms are introduced. Finally, a series of collected sample pictures are tested through the designed image algorithm to verify the feasibility and anti-interference of the algorithm.

## 2. Technical Scheme Design

**Figure 1:**
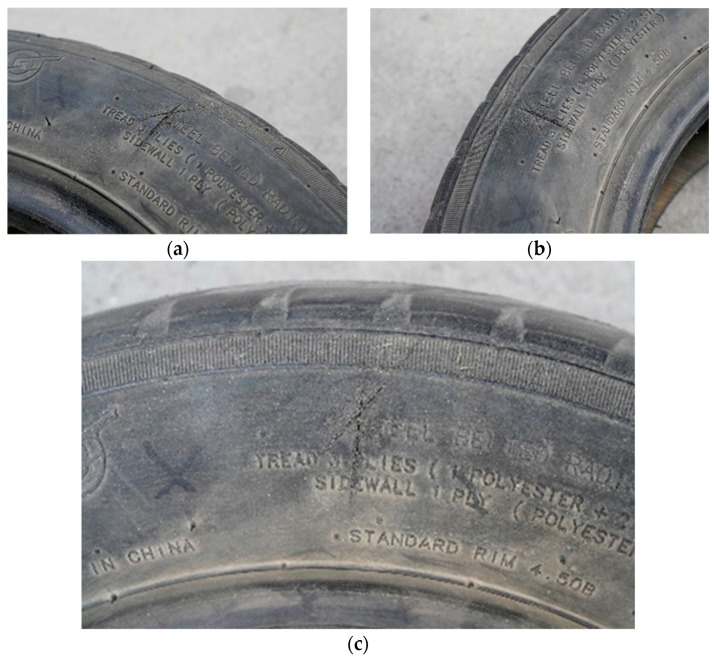
Sample demos. (**a**) The fifth sample of the total sample; (**b**) The 11th sample of the total sample; (**c**) The 13th sample of the total sample.

Tire damage is primarily caused by the horizontal or longitudinal friction of sharp objects on the road surface, resulting in various-shaped scratches. In this study, we choose to test the outer side of the tire. By utilizing a single micro-camera with a CMOS sensor, we can capture images of a specific wheel’s exterior and discard global tire vision. Through continuous local sampling of the tire side using wheel rotation (as shown in Figure 1), image processing technology is then employed to detect any damage present in the tested samples. Compared to global detection methods, although there is an increase in detection time, many irrelevant interferences are reduced, and algorithmic priors are improved. Consequently, detection efficiency is significantly enhanced while hardware requirements are simplified. These improvements enhance the feasibility of applying this research to engineering practice. In this study, we have selected the SONY A7M3 24-70G.28 single micro-camera (SONY (China) Co., LTD. Jinan branch, Jinan City, Shandong, China) as our image collection device due to its ability to meet all resolution requirements (6000 × 4000) for our detection index. All images were sampled under normal weather conditions.

**Figure 2:**
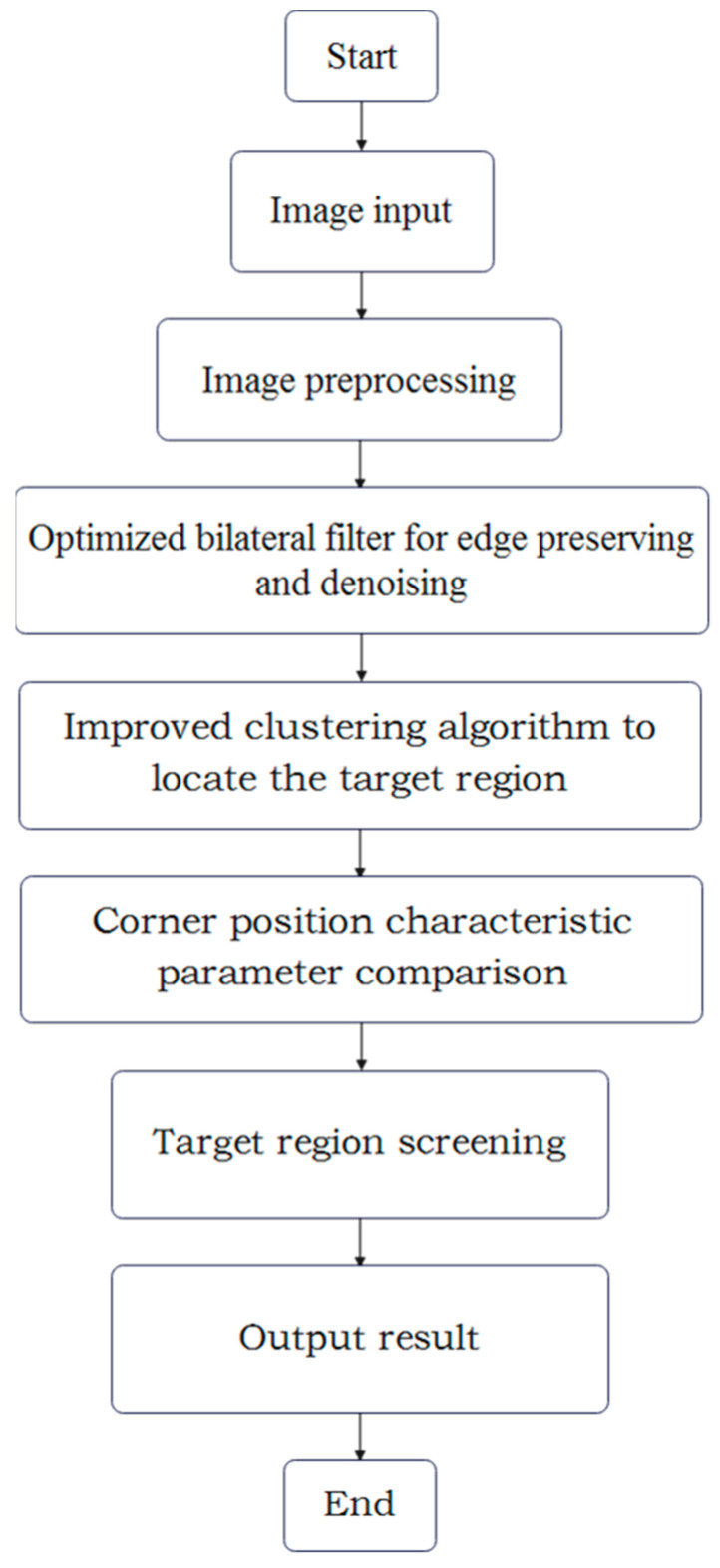
Flow chart of the proposed algorithm.

Tire damage is different from general visual inspection tasks. Through the method of collecting samples to be tested used in this paper, although the irrelevant interference caused by the structure of the car itself is avoided, the main interference is still distributed on the side surface of the tire, and it is not easy to find the damage site by naked eyes. As shown in Figure 1a, secondly, there is much non-damage irrelevant information such as the type of pressed tire and burr, so directly searching for the damage site is bound to produce a large number of false detections. In addition, the new and old degree of tire use will also bring a lot of interference information to the detection, which will not only increase the calculation amount of the detection, but also have a great impact on the subsequent positioning and screening. Since the color difference of the tire side is small, except for the background, some areas have the same color law, so before finding the scratch, it is necessary to highlight the characteristics of the scratch area and carry out a reasonable preprocessing algorithm for subsequent image segmentation. Due to the material characteristics of tire rubber, after damage, there will be little wear around the area, which will produce the “salt and pepper corners” shown in the image. The feature is captured and combined with noise for image filtering, which improves the contrast between the damaged area and the side surface of the tire, and enhances the edge information of the damaged area. Then, the image segmentation based on the improved clustering algorithm is used to locate the target area and non-target area. Finally, the target area judged as damaged is obtained by comparing the characteristic parameters. The flow chart of the algorithm is shown in Figure 2.

## 3. Image Segmentation Method Based on Improved K-Means Clustering Algorithm Validation

In order to detect the damage position in the image and solve the interference of irrelevant information, an image segmentation method based on improved clustering algorithm is designed in this paper, which mainly includes the following steps: firstly, image preprocessing is carried out, including optimized bilateral filtering and corner detection introduced in Section 4.1 below; finally, the improved clustering algorithm is applied to the filtered image. According to the color characteristics after clustering, the adaptive threshold is calculated to obtain the binary image to be matched and screened.

### 3.1. Image Preprocessing

The images captured by the image acquisition equipment typically consist of three channels representing RGB color information. Due to the relatively consistent tire color, distinguishing the noise in the tire tread poses a challenge. The noise exhibits the following characteristics: it appears extremely irregularly in terms of distribution and size within the image, with a majority of it being concentrated near areas of damage. Additionally, there is superposition and continuity observed in the noise present on the tread.

In general, traditional filtering methods are commonly used for image denoising, among which Gaussian filtering is a linear method. For noise points on grayscale images that belong to areas with significant changes in grayscale value, i.e., the high-frequency components, a Gaussian filter plays a role in smoothing the image and may introduce a “blurring” effect during the process. It exhibits strong denoising capabilities for noisy images. However, linear filters have limited applicability as they tend to blur edge information while achieving denoising effects. They not only struggle to enhance local contrast but also face challenges in extracting damaged areas. On the other hand, a median filter serves as a nonlinear alternative that can overcome some of these limitations by effectively removing noise while preserving edge information. Nevertheless, it often requires larger filter sizes which may result in loss of image details and subsequently affect the extraction process of damaged areas. Moreover, due to its characteristics and limitations as a linear filter, a Gaussian filter operates along only one direction whereas a median filter operates along both X and Y directions when using filters of equal size. Consequently, after reducing Gaussian noise with an identical filter size, the resulting image appears excessively smooth leading to fuzziness in target damage regions and poor retention of edge information. Although median filters excel at retaining edge information well, they significantly impact target damage regions.

**Figure 3:**
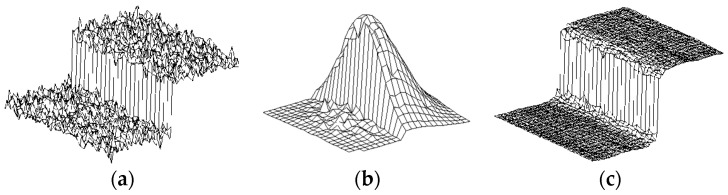
Schematic diagram of bilateral filtering principle. (**a**) Image noise distribution before bilateral filtering; (**b**) Edge protection and noise reduction; (**c**) Image noise distribution after bilateral filtering.

Therefore, this section introduces an optimized bilateral filtering method, which can further make the image achieve the preprocessing effect of “preserving edge”, “removing noise” and “preserving truth”. A bilateral filtering method is a nonlinear filtering method, which combines the compromise processing of spatial proximity and pixel value similarity, and considers the spatial information and grayscale similarity, so as to achieve the effect of edge preserving and denoising. Median filtering and mean filtering both belong to isotropic filtering, and they take the same attitude towards noise and edge information of the image. As a result, when the noise is smoothed out, the edges, textures and details that have an important role in the image are smoothed out at the same time, which is an undesirable result. In contrast, bilateral filtering can protect the edge well: that is, it can protect the edge characteristics of the image while removing noise, as shown in Figure 3, similar to “information retention between the steps, smoothing the blur inside the steps”.

Bilateral filtering can achieve both smooth denoising and good edge preservation, only because the kernel of a bilateral filter is generated by two functions: spatial domain kernel and pixel value domain kernel.

The optimized bilateral filtering algorithm introduced in this paper is actually a bilateral filtering based on multi-scale operation. The input image is split by a three-layer Laplacian pyramid method, and the low- and high-frequency filtering intensity is set, and the filtering is processed from the low-frequency layer to high-frequency layer in turn. According to the requirements of this paper, due to the existence of various irrelevant factors around the scratch damage of the tire side, it is necessary to better remove the surrounding noise while retaining the damage details: that is, increase the low-frequency filtering intensity and reduce the high-frequency filtering intensity.

In OpenCV, you can call the bilateral filter function to directly reduce the noise of the image, for the following input parameters:

(1) Size_d is used as the diameter range of the pixel neighborhood in the noise reduction process.
(2) Sigma_color is the σ value of the color space filter. The larger the value of this parameter, the wider colors in the pixel neighborhood will be mixed together to produce a larger semi-equal color region.
(3) Sigma_space is the σ value of the coordinate space filter, and the larger the value, the more distant pixels will affect each other, so that a larger area of similar enough colors can obtain the same color.

According to a large number of experiments, when size_d = 18, sigma_color = 120, sigma_space = 15, the image denoising effect is best based on the size of the image and the distribution characteristics of damaged and non-damaged areas. It is also applied to the multi-scale bilateral filtering process presented below.

**Figure 4:**
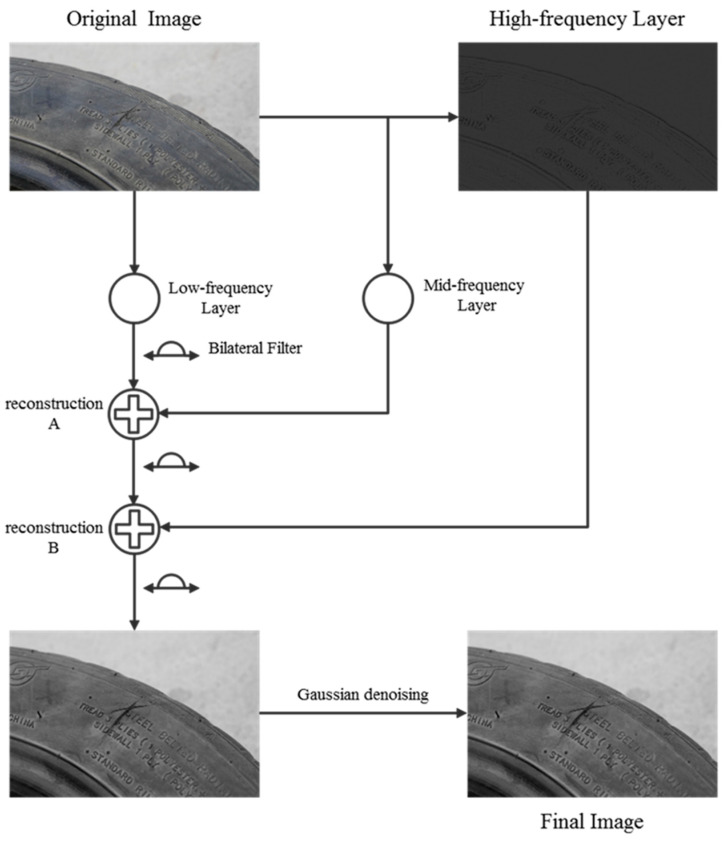
Multi-scale bilateral filter flow chart.

As shown in Figure 4, the original image is divided by the three-layer Laplacian pyramid method. The low-frequency layer is the part of the original image that changes slowly in grayscale, and the high-frequency layer is the part that changes fast in grayscale, which mainly highlights the edge information of the image, which can also be called the detail layer. As can be seen from the figure, the low-frequency image is firstly filtered by the original bilateral filter, and then reconstructed with the residuals of the mid-frequency layer to obtain reconstruction A; secondly, the reconstruction A is filtered bilaterally, and then the residual reconstruction with the high-frequency layer is carried out to reconstruct B; finally, the reconstruction B is filtered bilaterally again to get the final image output. In the process of reconstruction A, when the high-frequency layer is lost, the low-frequency part can achieve a good denoising effect and retain the details. After the reconstruction of B, the image regained the high-frequency layer, continued filtering, and enhanced the edge information of the image again to make the damaged area clearer. Finally, the final image was obtained by Gaussian smoothing and denoising.

**Figure 5:**
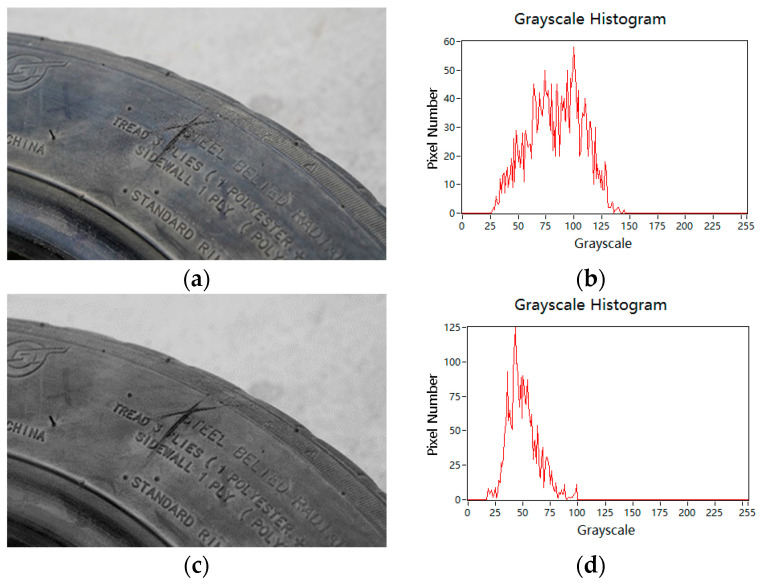
Comparison of blur results. (**a**) Original image; (**b**) Grayscale histogram of the original image; (**c**) After optimizing bilateral filtering; (**d**) Grayscale histogram of the Optimize bilateral filter.

Therefore, in this paper, the multi-scale bilateral filtering proposed above is used for image preprocessing. Figure 5. shows the experimental results compared with the original image after filtering and denoising. It can be seen from Figure 5b,d that the distribution of pixel gray values of the original image and the filtered image in locating the damaged area has a better effect on reducing the range of gray values due to the multi-scale bilateral filtering method: that is, the number of pixels with small gray values increases, thereby improving the contrast between the damaged area and the tread. The expected effect of smoothing and denoising the original image is achieved.

### 3.2. Principle of Clustering Algorithm

For RGB images, things with distinct color features can be recognized very quickly through color [10]. At the same time, the color feature is less dependent on the size, direction and perspective of the image itself, so the anti-interference ability is relatively strong [11,12]. In this paper, a method to obtain an adaptive threshold based on improved clustering algorithm is designed. Through the difference of gray value, it does not need a lot of details. After image clustering, the threshold value will be obtained according to the result [13,14], and the image segmentation will be carried out quickly, saving a lot of time.

The K-Means clustering algorithm divides the feature matrix X of a group of N samples into K clusters without intersection. Clusters are a group of aggregated data, and the data in a cluster can be considered to be the same kind of data, so the clusters are represented as the data results after clustering.

### 3.3. Image Segmentation Method under Improved K-Means Clustering

Image segmentation refers to the technology and process of dividing an image into several specific regions with unique properties and extracting the target of interest, which is the key step from image processing to image analysis [15]. 

Because the K-Means algorithm is very sensitive to the initial clustering center (centroid), different initial clustering centers will obtain different clustering effects. The function of the K-Means algorithm in OpenCV is to randomly select nodes as the initial clustering centers. Based on the characteristics of the image to be tested, this paper improves the simple and practical K-Means clustering algorithm and applies it to image segmentation, which can be divided into the following four steps:

(1) First, the first cluster center is randomly selected, which is the initial cluster center.
(2) Second, select the point far away from the first cluster center. The farther the distance, the higher the probability that it will be selected as the second cluster center. Thus, the target damage area with small gray value is different from the surrounding pixel with large gray value.
(3) Third, repeat the above operations until K center points are finally selected.
(4) Then, the cluster image is obtained, and the cluster including the target damage area is extracted to complete the image segmentation.

The above method is actually the K-Means++ algorithm. The K-Means++ algorithm is an improved version of the K-Means algorithm, proposed by David Arthur and Sergei Vassilvitskii in 2007. The K-Means++ algorithm is significantly better than the original K-Means algorithm, especially in the case of processing damaged image segmentation in this paper. 

In OpenCV 4.5.1, it happens to be possible to call the K-Means++ algorithm directly.

**Figure 6:**
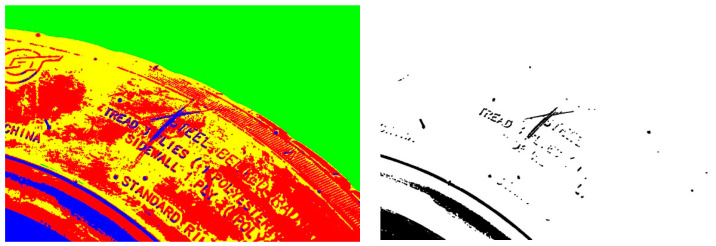
Experimental results of clustering image segmentation.

Figure 6 shows the final result of the improved target damage region in the clustering process. As can be seen from the figure, the blue region in the left figure is consistent with the binary segmentation image in the right figure. Although some irrelevant information is retained around the target region, the shape of the damage region is clearly visible, which lays a good foundation for the matching and screening below.

## 4. Matching Screening Method Based on Corner Detection

### 4.1. Corner Point

Corner points are very important features in the image [16,17]. While retaining important features of the image, these points can effectively reduce the amount of information data, make the information content very high, effectively improve the calculation speed, facilitate reliable image matching, and make real-time processing possible [18].

**Figure 7:**
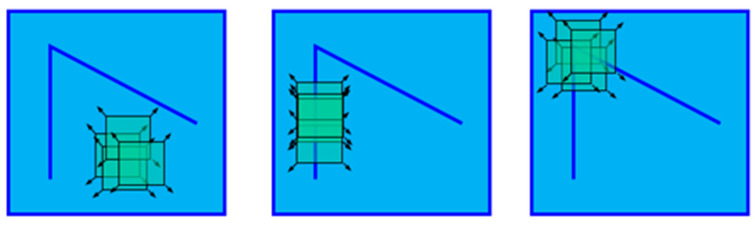
Corner characteristic diagram.

The Harris corner detection method is adopted in this paper; that is, the window slides on the image in any direction, as shown in Figure 7. If the window has no gray change in any direction, it is a flat area. The window has gray change in a certain direction; that is, the edge region. The window has obvious grayscale changes in any direction, so we can think that there are corner points in the window [19].

**Figure 8:**
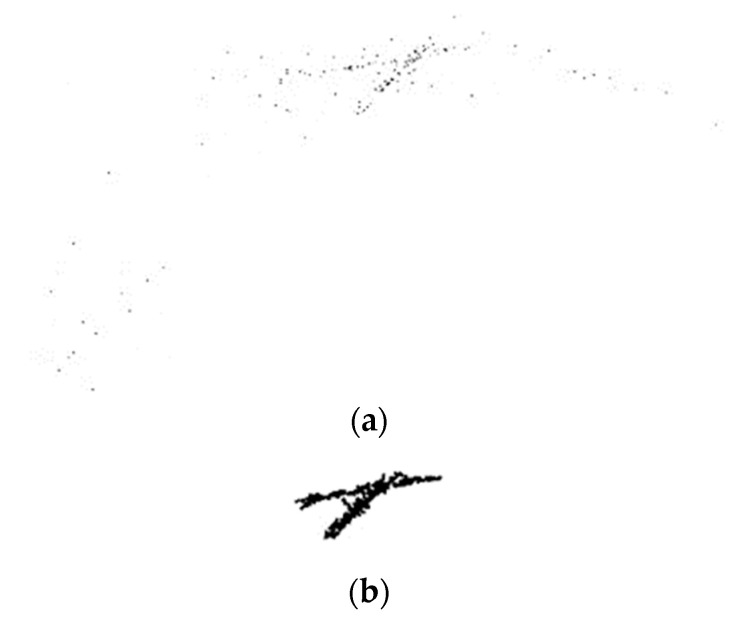
(**a**) Binarized image after capturing corner points; (**b**) Binarized image after capturing optimized.

According to the binary image results, the damaged area is basically preserved, because the figure includes the residual non-damaged area, which does not reach the final expected result, and the target area needs to be screened. In this paper, a matching screening method based on Harris corner detection is proposed. The main steps are as follows: Firstly, the “salt and pepper” corner detection is carried out on the original RGB image in the preprocessing stage, and the detected image is calculated by OTSU (Otsu method) to calculate the binary image. The practical significance is to highlight the corner points in the background of the detected image: as shown in Figure 8a, most of the corner points are concentrated in the damaged area. Finally, pixel increment and morphological optimization are carried out on the binary image to strengthen the corner points, and finally Figure 8b is obtained.

### 4.2. Matching Method Based on Histogram Correlation

The histograms of the two input images, Figure 8b and Figure 6, are calculated to obtain the histogram data H1 and H2, and normalized to obtain the same scale space. By calculating the similarity of the two groups of histogram data H1 and H2, the similarity of the image itself is compared. In OpenCV, by constructing and comparing the histogram model of the two images, the histogram similarity of the image is calculated, and 256 bins are set for screening, and the similarity value is judged. If the similarity value is greater than 0.90, the corner area and the damage area are successfully matched. The mark is output to obtain the damage area.

The shape of the corner combination can be obtained from the detection image results. It can be known from the above that the “salt and pepper corner” is distributed around the damage area, so the binarized image in Section 3.3 can be screened and matched through the obtained corner distribution shape map.

**Figure 9:**
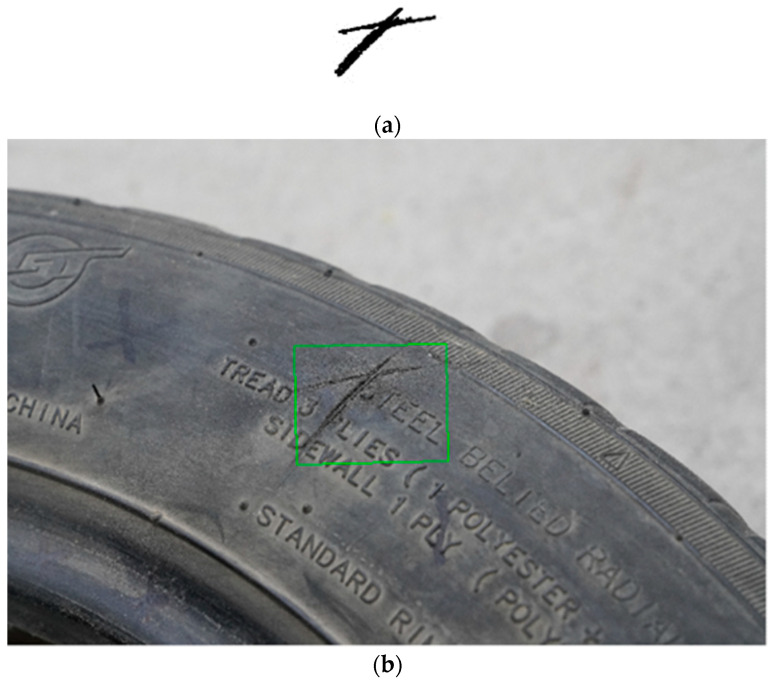
(**a**) Filter the matched target area; (**b**) The final experimental results.

As shown in Figure 9a, it can be seen from the screening results that the damaged area is completely matched. Since the same image size and format is used from beginning to end, the target area is marked by the matrix tool and displayed on the source image. The experimental results show that, as shown in Figure 9b, the region is completely selected, which proves the feasibility and effectiveness of the algorithm.

In order to verify the feasibility and anti-interference of the algorithm, a series of collected tire photos were tested and verified. In OpenCV, when the similarity value is equal to 1.00, it can be shown that the template diagram and the target diagram are almost the same. Input 30 images of tires with scratch damage in sequence. When the similarity is greater than 0.90, the output is True, and vice versa, the output is False. At the same time, the box selection algorithm identifies the scratch damage area with great similarity value. The final output is 30 times matrix box selection, and six groups of data are sampled according to random numbers, as shown in Table 1.

**Figure 10:**
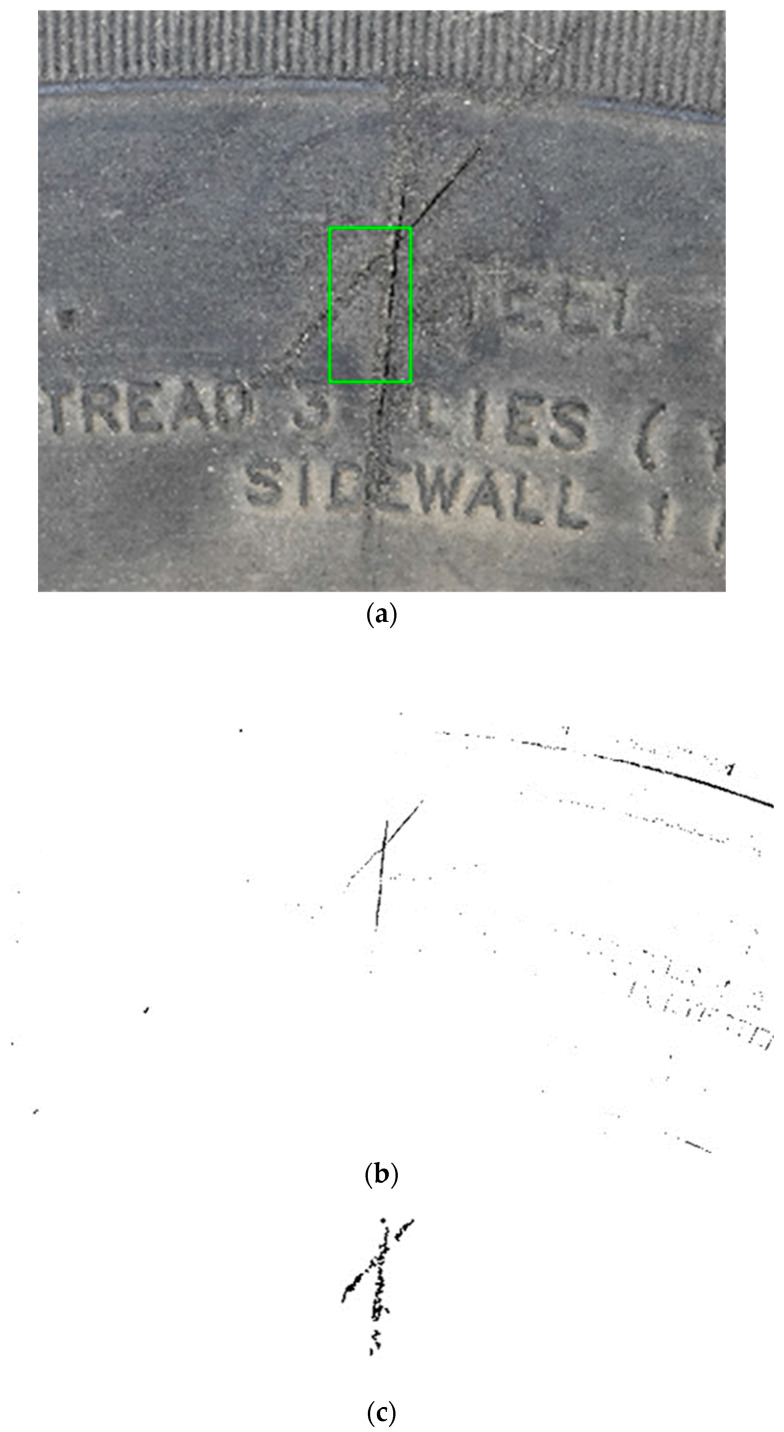
(**a**) Diagram of experimental anomaly results; (**b**) Pic. 13 Binary image; (**c**) Pic. 13 Corner point binarization image.

Among them, the maximum similarity value in Pic. 13 is abnormal to other numbers. After analyzing the output image, as shown in Figure 10a, it can be seen from the figure that the matrix tool did not completely select and mark the damaged area box, and then output its adaptive threshold to segment the image and capture the binarized image after corner point, as shown in Figure 10b,c. It is found that some regions of the corner point binarization image are discontinuous, resulting in a similarity value lower than 0.90, which makes the marking result abnormal. Experiments show that the algorithm is feasible.

## 5. Conclusions

The tire side damage detection method is investigated in this paper, focusing on image processing techniques. By employing a series of image preprocessing steps, segmentation algorithms, and effective matching and screening methods, the damaged area of the tire can be accurately extracted and successfully identified.

(1) This paper proposes a multi-scale bilateral filtering algorithm as a crucial component of image preprocessing. It effectively preserves edge information while achieving superior smoothing and denoising effects, thereby providing a solid foundation for subsequent image segmentation tasks. Based on the preprocessed images, an improved K-means clustering method is utilized to obtain an adaptive threshold for image segmentation. Consequently, a binary image representing the complete information of the damaged region is obtained. This approach offers high reliability and efficient performance.
(2) Based on the Harris corner detection method and combined with the characteristics around the tire damage area, the “salt and pepper” corner points in the wear zone in the edge of the area are captured, and the binary image of corner distribution is obtained, which accurately reflects the damage area. This method has anti-interference and provides convenience for subsequent matching screening and matrix box marking.
(3) In this paper, the region screening and matching based on histogram correlation method is used, the damage region can be accurately judged and matched by the maximum similarity, and the region matrix tool marker of the source image can be obtained by screening the edge corners. According to the test, the maximum value of the matching similarity can reach 1.00, and the remaining irrelevant information region can almost reach 0. It can meet the detection requirements of tire damage area.
(4) This paper explores the automatic detection method of tire tread damage. Although preliminary research results have been obtained, there are still limitations in the use environment. First, the material of the tire. The research on tire material and composition is relatively single, and this method cannot meet all the materials of tires on the market. Secondly, due to the small scale of the dataset and the few types of damage areas, it is more difficult for individuals to collect experimental data due to the particularity of the scratch damage problem. In addition, this method is mainly for tires with more tread damage, and there are more detection blind areas.

## Figures and Tables

**Table 1:** Random sampling test picture data table.

Picture Number	Max Similarity Value	Output
Pic. 3	0.9981	True
Pic. 11	0.9071	True
Pic. 13	0.6098	False
Pic. 21	0.9030	True
Pic. 27	1.0000	True
Pic. 30	1.0000	True

## Data Availability

Data are contained within the article.
